# Low Nitrogen Priming Enhances Photosynthesis Adaptation to Water-Deficit Stress in Winter Wheat (*Triticum aestivum* L.) Seedlings

**DOI:** 10.3389/fpls.2019.00818

**Published:** 2019-06-26

**Authors:** Jingwen Gao, Qiuci Luo, Chuanjiao Sun, Hang Hu, Feng Wang, Zhongwei Tian, Dong Jiang, Weixing Cao, Tingbo Dai

**Affiliations:** ^1^Key Laboratory of Crop Physiology Ecology and Production Management of Ministry of Agriculture, Nanjing Agricultural University, Nanjing, China; ^2^School of Biological Science, The University of Western Australia, Perth, WA, Australia; ^3^Environmental Resources and Soil Fertilizer Institute, Zhejiang Academy of Agricultural Sciences, Hangzhou, China

**Keywords:** drought, low nitrogen, photoinhibiton, photosynthesis, priming, stomatal, winter wheat

## Abstract

Drought is among the main environmental stressors that reduces wheat production. Nitrogen (N) availability affects plant adaptation to abiotic stress, but the effect of low N (LN) on drought tolerance is unclear. To identify the effect of LN priming on water-deficit stress tolerance in wheat seedlings, we primed cultivar Yangmai158 with 0.25 mM N for 7 days, and then added 20% polyethylene glycol 6000 as a water-deficit treatment for 5 days. The net photosynthetic rate (Pn), plant biomass, and plant growth rate (GR) were significantly reduced under water-deficit conditions; such decreases were less severe in LN-primed (LND) plants than non-primed (CKD) plants. The leaf relative water content (LRWC) decreased under water-deficit conditions, which in turn led to a reduced transpiration rate, stomatal conductance, and intercellular CO_2_ concentration (*C*_i_), causing a stomatal limitation on photosynthesis. LN priming also enhanced root growth, resulting in a higher LRWC and less stomatal limitation in LND plants than CKD plants. PSII quantum efficiency, photochemical quenching, and maximum PSII quantum efficiency were reduced under water-deficit conditions, indicating photoinhibition. However, LN priming increased the electron flux to photorespiration and the Mehler pathway, reducing photoinhibition. In conclusion, LN priming improved the leaf water status and increased alternative electron flux to attenuate photoinhibition, thus alleviating the inhibition of photosynthesis, and growth due to water deficiency.

## Introduction

Drylands cover about 45% of the Earth’s land surface and are home to more than 38% of the global population (Reynolds et al., 2007; Huang et al., 2016). Long-term global warming is predicted to increase soil drying because heating increases evaporation from plants and the soil surface (Trenberth et al., 2014; Cushman et al., 2015). The area of drylands is predicted to expand by 11–23% by the end of the 21st century to cover 50–56% of the global land surface by 2050 (Huang et al., 2016). Drought is among the main abiotic stressors restricting grain production worldwide (Chaves et al., 2002; Gallé et al., 2007; Varela et al., 2018). Crop production relies heavily on irrigation in drylands, with agriculture consuming more than 80% of the global fresh water supply (Hoekstra and Mekonnen, 2012; Cushman et al., 2015). Given that water scarcity is currently a serious problem worldwide, it is uncertain whether the water supply can meet agricultural production demands in the future (Jury and Vaux, 2005; Fereres and Soriano, 2006). Therefore, it is crucial to adopt proper water management practices to reduce irrigation water use. Improving the water-deficit stress tolerance of wheat seedlings would contribute to water conservation. Winter wheat (*Triticum aestivum* L.) is grown in arid and semi-arid regions. During vegetative growth stages, moderate drought causes negligible wheat yield reductions, whereas severe drought reduces yields significantly (Abid et al., 2016). Thus, reducing water input to wheat during vegetative growth stages could feasibly conserve water and guarantee grain yields.

Photosynthesis is an important process by which plants absorb light energy and assimilate CO_2_ to produce dry matter. Electrons generated from absorbed light energy pass through the electron transport chain to NADP^+^ (terminal electron acceptor), producing NADPH to provide reducing equivalents for the CO_2_ carboxylation process, and where NADP^+^ is regenerated to fuel electron transport (Warren, 2004). Within this highly interactive and regulated system, affecting one component causes changes to other components. Under water-deficit conditions, plants reduce their Tr to minimize water loss, which also leads to decreases in *G_s_* and *C_i_*s, directly restricting the photosynthetic CO_2_ carboxylation rate (Chaves, 1991; Yordanov et al., 2000). The reduction in CO_2_ carboxylation capacity under water-deficit stress may further lead to decreases in NADPH consumption and NADP^+^ regeneration rate, and the deletion of electron receptors results in the shutdown of PSII (Demmig-Adams and Adams, 1992; Niyogi, 1999). This PSII shutdown causes excess excitation energy to pass to oxygen, producing large amounts of free radicals, which further damage the photosynthetic apparatus and cause even worse impacts on growth by affecting the cytoarchitecture and other metabolic systems (Zivcak et al., 2013). Plants have several photoprotection strategies, including reducing the chlorophyll content and activity to cut down light energy absorption (Georgieva et al., 2009), increasing heat dissipation to dismiss excessive excitation energy (Flexas and Medrano, 2002), consuming excess excitation energy through alternative electron flux driven by photorespiration or the Mehler pathway (Miyake and Yokota, 2000; Zhou et al., 2004), and eliminating free radicals via the free radical scavenging system (Chaves and Oliveira, 2004). Therefore, maintaining a better leaf water status and improving the photoprotection capacity to sustain photosynthesis is important for improving plant water-deficit stress tolerance.

In our previous study, we concluded that the electron flux to photorespiration and the Mehler pathway increases under low nitrogen (LN) conditions, dissipating excess excitation energy and maintaining PSII function (Gao et al., 2018b). Therefore, strengthening the photoprotection capacity of plants through LN priming could play an important role in water-deficit stress tolerance. LN promotes root growth (Jiang et al., 2017), which can enhance water absorption under water-deficit stress, resulting in an improved leaf water status and increases in *G_s_* and the Pn. Zhang et al. (2016) found that reducing the N rate did not reduce wheat yields when basal N fertilizer application was postponed, and thus the N use efficiency was increased. Postponing basal N fertilizer application creates short-term LN conditions at the seedling stage, which can affect plant responses to later drought stress. Therefore, LN priming may improve water-deficit stress tolerance in wheat seedlings; such a finding would provide a theoretical basis for better water and N management.

In this study, a hydroponic experiment was conducted to study plant growth, root morphology, and photosynthesis responses to water-deficit stress in wheat seedlings after LN priming. We hypothesized that LN priming would enhance the water uptake capacity to increase *G_s_* and the CO_2_ carboxylation rate, and increase excess excitation energy consumption via photorespiration and the Mehler pathway to relieve photoinhibition caused by water-deficit stress.

## Materials and Methods

### Plant Materials and Experimental Design

**FIGURE 1:**
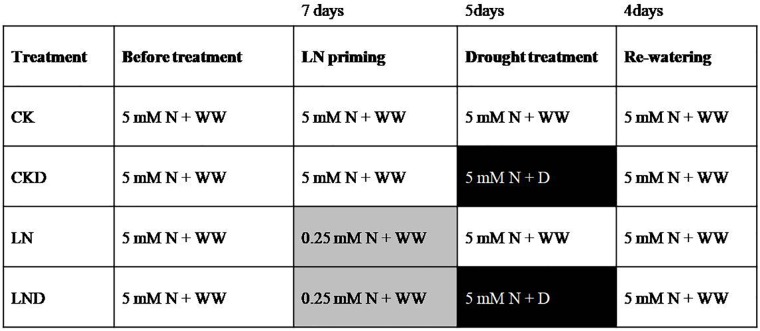
Experimental design for the study. Seedlings were grown in N-sufficient (5 mM N) and well water conditions (WW) before treatment (white background), and then divided into two groups, one grown in 0.25 mM N solution as an low nitrogen priming treatment (gray background), while the other grown in 5 mM N solution. After 7 days of low nitrogen priming, all the seedlings were grown in 5 mM N solution and half of plants in each treatment group were treated with 20% (m/v) polyethylene glycol 6000 (–0.6 MPa) to induce a water-deficit stress (D) (black background) for 5 days. After inducing water-deficit stress, the seedlings were re-watered for 4 days in 5 mM N solution.

The winter wheat cultivar Yangmai158 (seeds were reproduced by our lab and originally acquired from Jiangsu Academy of Agricultural Sciences) was planted for hydroponic experiments. Seeds of uniform size were surface-sterilized with 20% (v/v) H_2_O_2_ for 10 min, rinsed with distilled water, and then germinated in culture dishes covered with wet sterilized gauze in the dark. When the seed coleoptile length reached 1 cm, the plants were transferred to silica sand (soaked with 1% HCl for 2 days and flushed with copious amounts of water) and watered twice per day with distilled water. When the seedlings grew to the two-leaf stage, uniform seedlings were selected, and transplanted to opaque plastic containers filled with modified Hoagland nutrient solution (Gao et al., 2018a,b) under greenhouse conditions with a photoperiod of 16 h and a temperature cycle of 18°C (day)/8.5°C (night). The containers were 35 cm in length, 25 cm in width, and 10 cm in height. Each container contained 20 seedlings. Seedlings were first grown in N-sufficient (5 mM N) solution until the four-leaf stage, and then split into two batches, one grown in LN (0.25 mM N) solution for 7 days as an LN priming treatment, and the other grown in N-sufficient solution as a control. Half of the plants in each treatment group were subjected to a water deficit for 5 days by adding 20% (m/v) polyethylene glycol 6000 (–0.6 MPa) (Han et al., 2015) in N-sufficient solution; the remaining plants were grown in N-sufficient solution prepared with well water. Finally, all seedlings were re-watered for 4 days in N-sufficient solution. In total, four treatments were established: non-priming + non-water-deficit stress (CK), non-priming + water-deficit stress (CKD), priming + non- water-deficit stress (LN), and priming + water-deficit stress (LND). The detailed study design is provided in Figure 1.

The composition of the N-sufficient solution was as follows: 0.5 mM Ca(NO_3_)_2_, 2 mM KNO_3_, 1 mM (NH_4_)_2_SO_4_, 0.5 mM CaCl_2_, 0.5 mM CaSO_4_, 1 mM KH_2_PO_4_, 1 mM MgSO_4_, 0.5 mM NaCl, 10 M Fe-EDTA, 2.35 MH_3_BO_3_, 0.55 MMnSO_4_⋅H_2_O, 0.0385 MZnSO_4_⋅7H_2_O, 0.0165 MCuSO_4_⋅5H_2_O, and 0.0065 MH_2_MoO_4_. The composition of the low N solution was as follows: 0.075 mM Ca(NO_3_)_2_, 0.05 mM (NH_4_)_2_SO_4_, 0.25 mM K_2_SO_4_, 0.5 mM CaCl_2_, 0.5 mM CaSO_4_, 1 mM KH_2_PO_4_, 1 mM MgSO_4_, 0.5 mM NaCl, 10 MFe-EDTA, 2.35 MH_3_BO_3_, 0.55 MMnSO_4_⋅H_2_O, 0.0385 MZnSO_4_⋅7H_2_O, 0.0165MCuSO_4_⋅5H_2_O, and 0.0065 MH_2_MoO_4_. The nutrient solution was replaced every 3 days to keep the nutrient concentrations constant, aerated continuously, and adjusted with H_2_SO_4_ or NaOH to maintain the pH between 5.5 and 6.0. The experiment was arranged in a completely randomized block design. The placement of different treatments was randomized to control for edge effects in the greenhouse.

### Plant Sampling

Leaves and roots were sampled separately before water-deficit treatment, at 5 days after water-deficit treatment and at 4 days after rewatering. Three containers were harvested as three independent biological replicates at each time. For each replicate, ten plants in one container were mixed and first placed in liquid N for 12 h and then stored at –80°C for chemical analysis, while the other ten plants were mixed and dried for 20 min at 105°C and then at 75°C until constant weight. For each replicate, dry weight was obtained as average dry weight from ten plants. The GR after water-deficit treatment was calculated as follows:

$$\text{GR after water}-\text{deficit}=({\text{W}}_{\text{d}}-{\text{W}}_{0}^{})/5$$

where W_d_ is the plant dry weight after water-deficit treatment, and W_0_ is the plant dry weight before water-deficit treatment. The GR after rewatering was calculated as follows:

$$\text{GR after rewatering}=({\text{W}}_{\text{r}}-{\text{W}}_{\text{d}}^{})/4$$

where W_r_ is the plant dry weight after rewatering.

To determine leaf area, fresh leaves were scanned using a scanner (Epson 1680, Suwa, Japan) and finally analyzed using WinRHIZO Pro Vision 5.0 (Elmira, ON, Canada). Specific leaf area was determined by dividing leaf dry weight by leaf area. LRWC was determined according to Meng et al. (2013). Leaves were weighed immediately to obtain fresh weight (FW), soaked in water overnight in the dark, and weighed again to obtain turgid fresh weight (TW), and then dried at 75°C until constant weight (DW). LRWC was then calculated as follows:

LRWC=(FW−DW)/(TW−DW)

To determine the total root length, root surface area and root volume, root samples were scanned using a scanner (Epson 1680, Suwa, Japan) and finally analyzed using WinRHIZO Pro Vision 5.0 (Elmira, ON, Canada).

### Gas-Exchange Measurements

Net photosynthetic rate, intercellular CO_2_ concentration, and stomatal conductance were measured from 09:00 to 11:00 using a gas exchange system (Li-Cor 6400, Li-Cor Inc., United States). Leaf temperature during measurements was maintained at 25.0 ± 0.5°C, with steady photosynthetic PPFD of 1500 mol photons m^-2^ s^-1^. The reference CO_2_ in the cuvette was 400 ± 2.5 mol mol^-1^, the vapor pressure deficit (Vpdl) was at 1.1 ± 0.05 kPa, and the relative humidity was at 55–65%. Data were recorded after acclimating in the cuvette for at least 10 min. For each treatment, gas exchange measurements were determined on six plants.

### Chlorophyll Fluorescence Measurements

Chlorophyll fluorescence was measured using a Fluor-Imager (CF Imager, Technologia Ltd., Colchester, Essex, United Kingdom). The steady state fluorescence (*F_s_*) was determined under actinic light. Saturating light pulse (∼8000 mol photons m^-2^ s^-1^) was applied to obtain the maximum fluorescence under light-adapted (*F_m_’*) under actinic light. After removal of the actinic light and application of 3 s of far-red light, the minimal fluorescence of light-adapted state (*F_o_’*) was obtained. The minimum and maximum chlorophyll fluorescence (*F_o_* and *F_m_*) were determined after full dark adaptation for at least 30 min. The *Φ_PSII_* and maximum quantum efficiency of photosystem II (*F_v_/F_m_*) were calculated as $\frac{F_{\text{m}}{}^{\prime }-F_{\text{s}}}{F_{\text{m}}{}^{\prime }}$ and $\frac{F_{\text{m}}-F_{0}}{F_{\text{m}}}$, respectively (Genty et al., 1989). Photochemical quenching (*qL*, in the lake model) and NPQ was calculated as $\frac{F_{0}{}^{\prime }}{F_{\text{s}}}\times \frac{F_{\text{m}}{}^{\prime }-F_{\text{s}}}{F_{\text{m}}{}^{\prime }-F_{0}{}^{\prime }}$ and $\frac{F_{\text{m}}}{F_{\text{m}}{}^{\prime }}-1$, respectively (Kramer et al., 2004). Electron transport rate (*J_t_*) was calculated as $\frac{F_{\text{m}}{}^{\prime }-F_{\text{s}}}{F_{\text{m}}{}^{\prime }}$ × PPFD × 0.85 × 0.5 (Li et al., 2009).

### Calculation of the Electron Flux to Photosynthetic Carbon Reduction Cycle [Je(PCR)], Electron Flux to Photorespiratory Carbon Oxidation Cycle [Je(PCO)], and Alternative Electron Flux (Ja)

Photosynthetic rate (A) is mathematically expressed as:

$$\text{A}=v_{c}-0.5v_{0}-R_{\text{d}}=v_{c}\times \left(1-\frac{\Gamma *}{C_{i}}\right)-R_{\text{d}}$$

where *v_c_* is the Rubisco carboxylation rate, *v_o_* is the Rubisco oxygenation rate, *R*_d_ is the mitochondrial respiration rate in the light, and *Γ*^∗^ is the CO_2_ compensation point related to the *C*_i_ which was measured according to the method of Li et al. (2009). Then, *v_c_* and *v_o_* can be calculated.

The electron fluxes in the photosynthetic carbon reduction cycle, photorespiratory carbon oxidation cycle, and alternative electron flux are expressed as follows (Zhou et al., 2004):

$$\begin{array}{c} Je(PCR)=4\times v_{c}\\ Je(PCO)=4\times v_{0}\\ Ja=Jt-Je(PCR)-Je(PCO) \end{array}$$

### Determination of O_2_^-^ Production Rate and Malondialdehyde (MDA) Concentration

Frozen leaves (0.5 g) were homogenized in 5 cm^3^ of ice cold 5 mM sodium phosphate buffer (50 mM, pH 7.0) containing 0.5 mM EDTA, 10 mM MgCl_2_. The mixture was centrifuged at 10000 g (4°C) for 15 min. The supernatant was used to determine O_2_^-^ production rate and MDA concentration according to Liu et al. (2017).

### Statistical Analysis

The significance of the effects of LN priming and water-deficit at the 0.05 level was determined by two-way analysis of variance (ANOVA) using SPSS 19.0 software (SPSS, Inc., Chicago, IL, United States). Principal component analysis (PCA) was performed by using the data of all measured physiological parameters using prcomp() in R^1^. Graphs were plotted using Sigmaplot 10.0 software (Systat Software, Inc., Chicago, IL, United States).

## Results

### Dry Weight and Leaf Area

**FIGURE 2:**
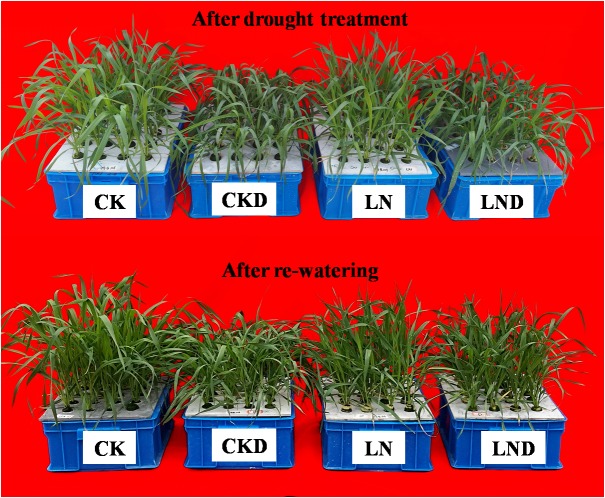
Effect of LN priming on plant growth of wheat seedlings after water-deficit treatment and rewatering.

**FIGURE 3:**
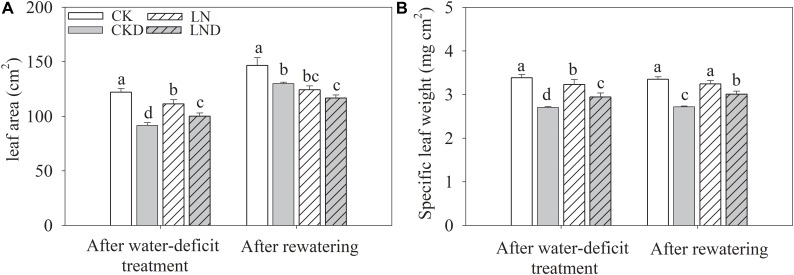
Effect of LN priming on leaf area **(A)** and specific leaf weight **(B)** of wheat seedlings after water-deficit treatment and rewatering. Data were expressed as means over three replicates. Lowercase letters indicates significant difference at the 0.05 level among four treatments after either water-deficit treatment or rewatering, which were determined by Dunnett’s multiple comparison test.

Compared with CK, LN treatment resulted in decreases in shoot and plant (shoot + root) biomass, GR (Figure 2 and Table 1), leaf area (Figure 3A), and specific leaf weight (Figure 3B), and an increase in root biomass. Water-deficit treatment (CKD and LND) significantly decreased the shoot and plant biomass, GR, leaf area, and specific leaf weight. However, these parameters were higher in LND plants than CKD plants, indicating that growth inhibition due to water-deficit stress was relieved by LN priming. After 5 days of re-watering, the GR in LND plants recovered to LN levels, whereas that in CKD plants was still lower than in CK plants. GR, plant biomass, and specific leaf weight were significantly higher in LND plants than in CKD plants.

**Table 1:** Effects of low nitrogen priming on dry weight of wheat seedlings after water-deficit treatment and rewatering.

	Treatment	Shoot dry weight (g plant^-1^)	Root dry weight (g plant^-1^)	Plant dry weight (g plant^-1^)	Plant growth rate (GR) (g plant^-1^ day^-1^)
After	CK	0.610 ± 0.016a	0.111 ± 0.001b	0.721 ± 0.017a	0.039 ± 0.002a
water-deficit	CKD	0.431 ± 0.005d	0.106 ± 0.007b	0.538 ± 0.006d	0.002 ± 0.001d
treatment	LN	0.528 ± 0.006b	0.139 ± 0.003a	0.667 ± 0.008b	0.033 ± 0.001b
	LND	0.466 ± 0.007c	0.143 ± 0.004a	0.609 ± 0.007c	0.022 ± 0.002c
After rewatering	CK	0.731 ± 0.014a	0.154 ± 0.001b	0.885 ± 0.015a	0.041 ± 0.001a
	CKD	0.534 ± 0.004c	0.119 ± 0.004c	0.654 ± 0.002d	0.029 ± 0.002c
	LN	0.620 ± 0.007b	0.203 ± 0.004a	0.823 ± 0.008b	0.039 ± 0.001b
	LND	0.556 ± 0.011c	0.203 ± 0.010a	0.760 ± 0.007c	0.038 ± 0.001b

*Data were expressed as means ± SE, n = 3. Lowercase letters following the data within the same column refer to significant differences (P < 0.05), which were determined by Dunnett’s multiple comparison test.*

### Leaf Relative Water Content (LRWC)

**FIGURE 4:**
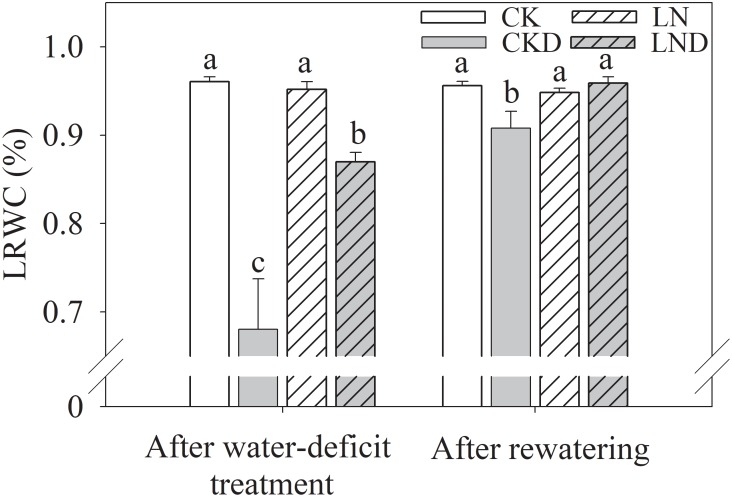
Effects of LN priming on LRWC of wheat seedlings after water-deficit treatment and rewatering. Data were expressed as means over three replicates. Lowercase letters indicates significant difference at the 0.05 level among four treatments after either water-deficit treatment or rewatering, which were determined by Dunnett’s multiple comparison test.

As shown in Figure 4, there was no significant difference in LRWC between CK and LN plants. Water-deficit treatment (CKD and LND) significantly reduced the LRWC compared with CK. However, the LRWC was significantly higher in LND plants than in CKD plants. After 5 days of re-watering, the LRWC in LND plants recovered to CK and LN levels, whereas the LRWC remained significantly lower in CKD plants than in CK plants.

### Root Morphology

Compared with CK, LN treatment significantly increased the total root length, root surface area, and root volume (Table 2). In contrast, the total root length, root surface area, and root volume decreased significantly in CKD plants compared with CK plants; however, that in LND plants did not decrease relative to LN plants and was significantly higher than that in CK and CKD plants. After re-watering, the total root length, root surface area, and root volume were higher in LND plants than in CK and LN plants, whereas that in CKD plants remained lower than in CK plants.

**Table 2:** Effects of LN priming on root length, root surface area and root volume of wheat seedlings after water-deficit treatment and rewatering.

	Treatment	Total root length (cm)	Root surface area (cm^2^)	Root volume (cm^3^)
After	CK	2393 ± 126 b	225 ± 6 b	1.74 ± 0.12 b
water-deficit	CKD	1885 ± 149 c	161 ± 12 c	1.22 ± 0.04 c
treatment	LN	3052 ± 206 a	285 ± 11 a	2.04 ± 0.08 a
	LND	3019 ± 182 a	291 ± 2 a	2.02 ± 0.05 a
After rewatering	CK	3260 ± 53 c	296 ± 8 b	2.14 ± 0.12 b
	CKD	2208 ± 45 d	186 ± 11 c	1.35 ± 0.07 c
	LN	3536 ± 79 b	310 ± 9 b	2.22 ± 0.15 b
	LND	3972 ± 162 a	357 ± 9 a	2.46 ± 0.13 a

*Data were expressed as means ± SE, n = 3. Lowercase letters following the data within the same column refer to significant differences (P < 0.05), which were determined by Dunnett’s multiple comparison test.*

### Gas Exchange Parameters

**FIGURE 5:**
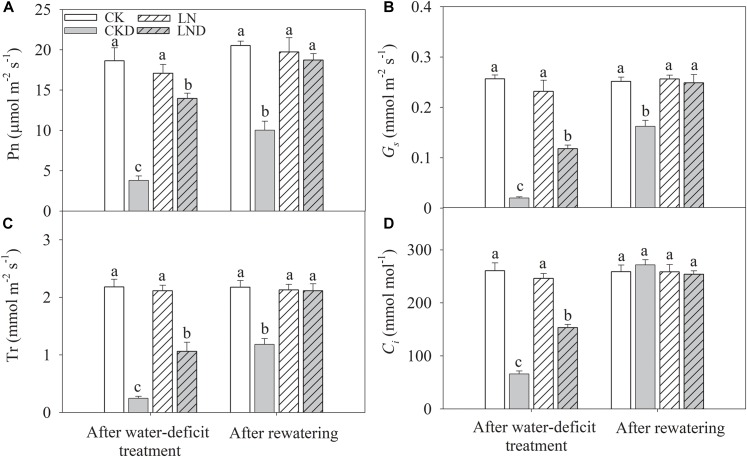
Effect of LN priming on Pn **(A)**, *G_s_* **(B)**, Tr **(C)**, and *C_i_* **(D)** of wheat seedlings after water-deficit treatment and rewatering. Data were expressed as means over three replicates. Lowercase letters indicates significant difference at the 0.05 level among four treatments after either water-deficit treatment or rewatering, which were determined by Dunnett’s multiple comparison test.

There were no significant differences in Pn (Figure 5A), *G_s_* (Figure 5B), Tr (Figure 5C), and *C_i_* (Figure 5D) between CK and LN plants. Under water-deficit conditions, Pn, Tr, *G_s_*, and *C_i_* were significantly lower in CKD and LND plants than in CK plants. However, Pn, Tr, *G_s_*, and *C_i_* were significantly higher in LND plants than in CKD plants. After 5 days of re-watering, these gas exchange parameters in LND plants increased to CK and LN levels, whereas those in CKD plants remained significantly lower than CK levels.

### Photoinhibition and Photoprotection

**FIGURE 6:**
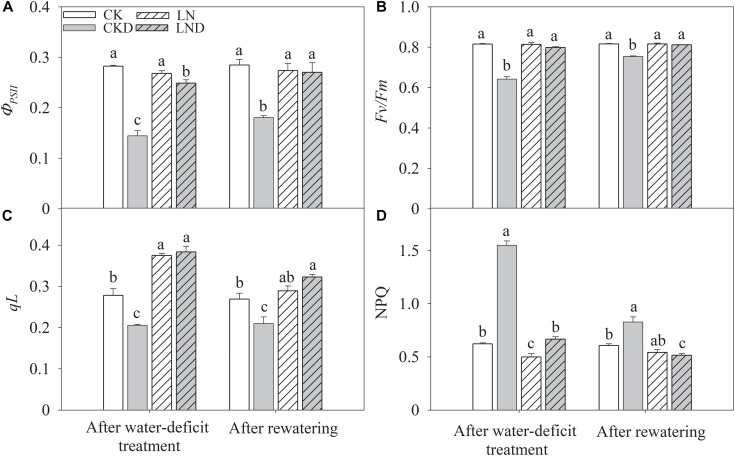
Effect of LN priming on *Φ_PSII_* **(A)**, *F_v_/F_m_* **(B)**, *qL* **(C)**, and NPQ **(D)** of wheat seedlings after water-deficit treatment and rewatering. Data were expressed as means over three replicates. Lowercase letters indicates significant difference at the 0.05 level among four treatments after either water-deficit treatment or rewatering, which were determined by Dunnett’s multiple comparison test.

Compared with CK, LN treatment had no significant effects on PSII efficiency (*Φ_PSII_*) (Figure 6A), and the ratio of variable fluorescence to maximal fluorescence (*F_v_/F_m_*) (Figure 6C); however, *qL* (Figure 6B) increased and NPQ (Figure 6D) decreased. Under water-deficit conditions, *Φ_PSII_* decreased significantly in CKD and LND plants compared with CK plants, but *Φ_PSII_* was higher in LND plants than in CKD plants. Both *qL* and *F_v_/F_m_* were lower in CKD plants than in CK plants, whereas the values of these parameters did not decrease in LND plants compared with CK and LN plants. In contrast, water-deficit treatment (CKD and LND) significantly increased NPQ, which was higher in CKD plants than in LND plants. After 5 days of re-watering, *Φ_PSII_* recovered in LND plants to LN and CK levels, whereas *Φ_PSII_*, *qL*, and *F_v_/F_m_* remained lower and NPQ higher in CKD plants compared with CK plants.

**FIGURE 7:**
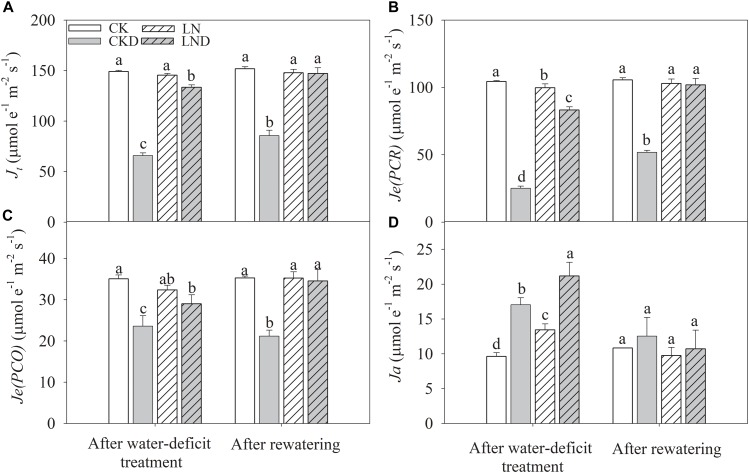
Effect of LN priming on *J_t_* **(A)**, *Je(PCR)* **(B)**, *Je(PCO)* **(C)**, and *Ja* **(D)** of wheat seedlings after water-deficit treatment and rewatering. Data were expressed as means over three replicates. Lowercase letters indicates significant difference at the 0.05 level among four treatments after either water-deficit treatment or rewatering, which were determined by Dunnett’s multiple comparison test.

Compared with CK plants, *Je(PCR)* (Figure 7B) decreased in LN plants, but *Ja* (Figure 7D) increased, whereas *Je(PCO)* (Figure 7C) and J_t_ (Figure 7A) remained unchanged. Under water-deficit conditions, *J*_t_*, Je(PCR)*, and *Je(PCO)* decreased significantly in CKD and LND plants compared with CK plants. However, *J*_t_*, Je(PCR)*, and *Je(PCO)* were significantly higher in LND plants than in CKD plants. In contrast, *Ja* increased under water-deficit conditions, and was higher in LND plants than in CKD plants. After 5 days of re-watering, there were no significant differences in *J*_t_*, Je(PCR), Je(PCO)*, and *Ja* among the LND, LN, and CK plants, whereas *J*_t_*, Je(PCR)*, and *Je(PCO)* remained significantly lower in CKD plants than in CK plants.

### Free Radicals

**FIGURE 8:**
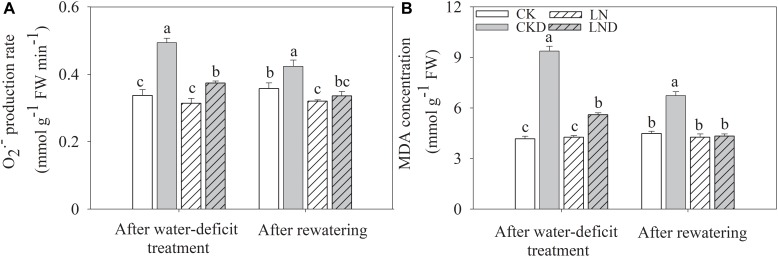
Effect of LN priming on O_2_^-^ production rate **(A)** and MDA concentration **(B)** of wheat seedlings after water-deficit treatment and rewatering. Data were expressed as means over three replicates. Lowercase letters indicates significant difference at the 0.05 level among four treatments after either water-deficit treatment or rewatering, which were determined by Dunnett’s multiple comparison test.

There were no significant differences in the O_2_^-^ production rate (Figure 8A) and MDA concentration (Figure 8B) between CK and LN plants (Figure 8). Under water-deficit conditions, these parameters were higher in CKD and LND plants than in CK plants, but lower in LND plants than in CKD plants. After re-watering, the O_2_^-^ production rate and MDA concentration in LND plants reduced to CK and LN levels, whereas those in CKD plants remained higher than in CK plants.

### PCA of Growth and Physiological Parameters

**FIGURE 9:**
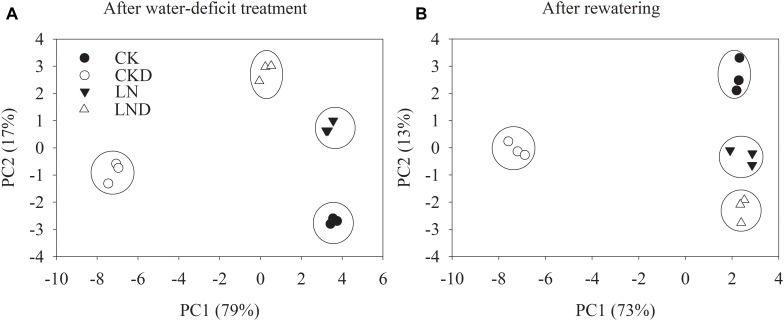
PCA of growth and physiological traits under combined conditions of water and N after water-deficit treatment **(A)** and rewatering **(B)**. Growth traits and physiological parameters are included in PCA.

To unravel key parameters involved in the response patterns of both non-LN primed plants and LN primed plants to water-deficit stress, a PCA was conducted using data of morphological and physiological parameters. After water-deficit treatment, PC1 and PC2 accounted for 79 and 17% of the variation, respectively (Figure 9A). PC1 clearly separated the effects of water-deficit treatment, and PC2 uncovered the effects of LN priming. Plant dry weight, plant GR, specific leaf weight, LRWC, Pn, *G_s_*, *C_i_*, Tr, *Φ_PSII_*, *F_v_/F_m_*, NPQ, *J*_t_, *Je(PCR)*, *Je(PCO)*, O_2_^-^ production rate, and MDA concentration were key contributors to PC1, whereas shoot dry weight, root dry weight, root total length, root surface, root volume, leaf area, *qL* and *Ja* were important factors to PC2 (Table 3).

**Table 3:** PCA of growth and physiological parameters under combined conditions of water and N.

	After water-dificit treatment		After rewatering	
	PC1	PC2	PC1	PC2
Shoot dry weight	0.188	-**0.2691**	0.1324	**0.4615**
Root dry weight	0.1068	**0.4183**	0.197	-**0.2783**
Plant dry weight	**0.2133**	-0.1644	**0.2014**	**0.2971**
Plant growth rate	**0.2257**	-0.0766	**0.2267**	0.1478
Root total Length	0.1575	**0.3315**	**0.2176**	-**0.2022**
Root surface	0.1675	**0.3244**	**0.2211**	-0.1842
Root volume	0.1872	**0.2627**	**0.2195**	-0.1504
Leaf area	0.1974	-**0.2246**	-0.0113	**0.5475**
Specific leaf weight	**0.2086**	-0.1633	0.1995	**0.2751**
LRWC	**0.2239**	0.0025	-0.0447	0.0614
Pn	**0.2275**	-0.0107	**0.2292**	0.0794
*G_s_*	**0.2196**	-0.128	**0.2321**	0.0131
*C_i_*	**0.2222**	-0.1084	-0.1423	0.0693
Tr	**0.2199**	-0.1097	**0.2327**	0.041
*Φ_PSII_*	**0.2271**	0.0232	**0.2267**	0.0952
*qL*	0.1549	**0.3502**	**0.2055**	-**0.2461**
*F_v_/F_m_*	**0.2234**	0.0881	**0.2374**	0.0372
NPQ	-**0.2229**	-0.1138	-**0.227**	0.1429
*J_t_*	**0.2269**	0.0439	**0.2373**	0.0458
*Je(PCR)*	**0.2288**	0.0111	**0.2374**	0.0462
*Je(PCO)*	**0.2083**	-0.1289	**0.2334**	0.041
*Ja*	-0.1141	**0.3915**	-0.1135	-0.0129
O_2_^-^ production rate	-**0.2249**	-0.0462	-**0.2177**	0.1088
MDA	-**0.2293**	-0.0138	-**0.2357**	0.0226

*The numbers in bold fonts indicate key factors of PC1 and PC2 with abstract of scores larger than 0.20.*

After rewatering, PC1 and PC2 accounted for 73 and 13% of the variation, respectively (Figure 9B). Plant dry weight, plant GR, root total length, root surface, root volume, Pn, *G_s_*, *C_i_*, Tr, *Φ_PSII_*, *qL*, *F_v_/F_m_*, NPQ, *J*_t_, *Je(PCR)*, *Je(PCO)*, O_2_^-^ production rate, and MDA concentration were key contributors to PC1, whereas shoot dry weight, root dry weight, plant dry weight, root total length, leaf area, specific leaf area, and *qL* were important factors to PC2.

## Discussion

Drought has been widely reported as the main abiotic stressor constraining wheat yields, affecting leaf water relations and photosynthesis, resulting in limited GRs (Chaves et al., 2002; Gallé et al., 2007; Varela et al., 2018). Consistent with previous studies, our results show that leaf area (Figure 3A) and Pn (Figure 5A) decreased under water-deficit stress in wheat seedlings, leading to a reduced GR and dry matter (Table 1). After 5 days of rewatering, the GR had not recovered. Proper cultivation management can be applied to improve crop water-deficit stress tolerance, thereby minimizing the effects of water deficiency on plant growth, yield, and quality. N, the most abundant element in the atmosphere, is essential for plant growth, and it strongly affects plant productivity. A reduced N supply leads to severe plant growth inhibition and lower grain yields (Ladha et al., 2005; Lu et al., 2015). In our previous study, we concluded that short-term (6 days) LN treatment had negligible effects on wheat seedling growth, but induced an increase in Pn (Gao et al., 2018a). In the present study, despite a decrease in plant biomass due to LN priming, water-deficit stress-induced reductions in Pn, GR, and plant dry weight occurred to a smaller extent in LND plants compared with CKD plants, and the GR of LND plants was significantly higher than that of CKD plants after 4 days of re-watering. These results indicate that LN priming improved water-deficit stress tolerance in wheat seedlings.

Under leaf water deficiency, plants reduce stomatal opening to decrease water loss through transpiration; however, this response leads to decreased *G_s_* (Figure 5B) and *C_i_* (Figure 5D), restricting photosynthetic CO_2_ carboxylation and directly causing a reduction in Pn (Grassi and Magnani, 2005). We showed that the LRWC (Figure 4), Tr (Figure 5C), *G_s_*, and *C_i_* decreased under water-deficit stress (Figure 5). However, compared with CKD plants, LND plants maintained a higher LRWC, Tr, *G_s_*, and *C_i_*. Developed root systems increase water absorption; a previous study reported decreased cytokinin (CTK) synthesis and increased indole-3-acetic acid (IAA) synthesis under LN conditions, resulting in high IAA/CTK ratios, which benefit root growth (Zhang et al., 2007). In the present study, after LN priming, root dry weight, total root length, root surface, and root volume improved significantly (Table 2), consistent with the results of previous studies (Hermans et al., 2006; Jiang et al., 2017) that reported better leaf water status, sustained transpiration, and stomatal opening. Better root water absorption ability accelerates the recovery of leaf water status after re-watering. After 4 days of re-watering, the LRWC, Tr, and *G_s_* in LND plants recovered to CK and LN levels, whereas those of CKD plants remained lower than those of CK plants. Therefore, improved root growth induced by LN priming resulted in higher water absorption ability, which led to better leaf water status and maintained transpiration, stomatal opening, and photosynthesis, benefiting growth recovery after re-watering.

After 4 days of re-watering, *C_i_* in CKD plants recovered to CK levels, whereas Pn in CKD plants remained lower than that in CK plants, suggesting that photosynthesis was also restricted by non-stomatal factors under water-deficit conditions. Previous studies have reported that water-deficit stress leads to an imbalance between light energy harvesting and utilization during the CO_2_ assimilation process (Galmés et al., 2007; Khanna-Chopra and Selote, 2007), in turn shutting down PSII and blocking photosynthetic electron transport (Demmig-Adams and Adams, 1992; Niyogi, 1999). Our findings show that water-deficit stress shut down PSII, indicated by a decrease in *qL* (Figure 6B; Maxwell and Johnson, 2000). As a result, *ΦPSII* (Figure 6A) and *J_t_* (Figure 7A) decreased and NPQ (Figure 6D) increased, suggesting that more light energy was dissipated as heat. After 4 days of re-watering, NPQ remained higher and *ΦPSII* and *J_t_* lower in CKD plants than in CK plants. Although dissipating excess absorbed light energy as heat is an important photoprotection approach, this protective dissipation reduces photosynthetic efficiency and potential yields, especially when it does not decrease rapidly after stress is removed (Kromdijk et al., 2016). When absorbed light energy exceeds dissipation capacity, spare electrons trigger the production of reactive oxygen species, damaging the photosynthetic apparatus (Pascal et al., 2005). Our results show that under water-deficit conditions, the O_2_^-^ production rate (Figure 8A) and MDA concentration (Figure 8B) increased significantly (Figure 8) and *F_v_/F_m_* (Figure 6C) decreased significantly, indicating photodamage. However, reductions in *qL*, *ΦPSII*, and *J_t_* and increases in NPQ, O_2_^-^ production rate, and MDA concentration were lower in LND plants than in CKD plants, and *F_v_/F_m_* did not decrease in LND plants. After 4 days of re-watering, the Pn, chlorophyll fluorescence parameters, and free radical levels in LND plants recovered to CK and LN levels. These results suggest that water-deficit stress led to photoinhibition, but that it was relieved by LN priming.

Intercellular CO_2_ concentration increased in LND plants compared with CKD plants, resulting in higher *Je(PCR)* (Figure 7B) and Pn, and thus more excitation energy being consumed in the CO_2_ assimilation process. However, photorespiration and the Mehler pathway (water–water cycle) play important roles in consuming excess excitation energy, maintaining PSII function, and sustaining electron transport (Niyogi, 1999; Miyake and Yokota, 2000; Zhou et al., 2004). In our previous study, we concluded that the photorespiratory and Mehler pathways were enhanced under LN conditions (Gao et al., 2018b). In the present study, *Je(PCO)* (Figure 7C) and *Ja* (Figure 7D) were significantly higher in LND plants than in CKD plants, suggesting that the ability to dissipate excitation energy through these pathways was improved after LN priming. Therefore, the ability to dissipate excess excitation energy via CO_2_ assimilation and the photorespiratory and Mehler pathways was improved by LN priming, which played an important role in photoprotection under water-deficit stress.

Principal component analysis (Figure 9 and Table 3) demonstrated that the effects of water-deficit treatment were clearly separated by PC1, while the effect of LN priming was uncoupled by PC2. PC1 was higher than PC2, indicating the water-deficit treatment had greater effect on morphological and physiological parameters than LN priming. The key factors in PC1 were plant dry weight, plant GR, specific leaf weight, LRWC, Pn, *G_s_*, *C_i_*, Tr, *Φ_PSII_*, *F_v_/F_m_*, NPQ, *J*_t_, *Je(PCR)*, *Je(PCO)*, O_2_^-^ production rate and MDA concentration, indicating that water-deficit stress largely impacted plant growth, photosynthesis and production of reactive oxygen species. The key factors in PC2 were shoot dry weight, root dry weight, root total length, root surface, root volume, leaf area, *qL* and *Ja*, indicating that LN priming influenced root growth and photoprotection process. Moreover, in the PCA plot, a greater distance between symbols related to between CK and CKD to that between LN and LND indicated that non-LN primed plants were more sensitive to water-deficit stress than LN primed plants.

In conclusion, LN priming maintained stomatal opening by improving water absorption ability due to a developed root system, and protected the photosynthetic apparatus by consuming excess excitation energy through CO_2_ assimilation and alternative electron flux, which contributed to sustained photosynthesis and plant growth under water-deficit stress and promoted growth recovery after re-watering. However, the present study was conducted only under hydroponic conditions and lacked yield results. Therefore, whether LN priming can reduce yield losses caused by drought during seedling growth remains to be confirmed in field experiments, which will provide a theoretical basis for water and N-saving cultivation.

## Author Contributions

JG, QL, and TD planned and designed the research. JG, QL, and CS conducted the experiments and fieldwork, and analyzed the data. JG, HH, FW, ZT, DJ, WC, and TD wrote the manuscript.

## Conflict of Interest Statement

The authors declare that the research was conducted in the absence of any commercial or financial relationships that could be construed as a potential conflict of interest.

## Footnotes

1 http://www.r-project.org/

## Abbreviations

*C*_i_: intercellular CO_2_ concentration
F_v_/F_m_: maximum quantum efficiency of PSII
GR: growth rate
*Gs*: stomatal conductance
*Ja*: alternative electron flux
*Je(PCO)*: electron fluxes to photosynthetic carbon reduction
*Je(PCR)*: electron fluxes to photorespiratory carbon oxidation
*J_t_*: rate of electron transport through PSII
LRWC: leaf relative water content
MDA: malondialdehyde
NPQ: non-photochemical quenching
PPFD: photon flux intensity
Pn: net photosynthetic rate
PSII: photosystem II
*qL*: photochemical quenching
Tr: transpiration rate
*Γ*^∗^: the CO_2_ compensation point related to Ci
φ: apparent quantum yield
Φ_PSII_: quantum efficiency of PSII
